# Liver-Derived Peptides from Livestock Processing By-Products: Insights into Generation, Bioactivity, and Taste Properties

**DOI:** 10.3390/foods15142497

**Published:** 2026-07-14

**Authors:** Zhilin Chen, Ahmed H. Abdullah, Wei Wu, Yu Fu

**Affiliations:** 1 College of Food Science, Southwest University, Chongqing 400715, China; baiht333@163.com (Z.C.); a.hassan@agr.svu.edu.eg (A.H.A.); 2 Westa College, Southwest University, Chongqing 400715, China; 3 Department Food Science and Technology, Faculty of Agriculture, Qena University, Qena 83523, Egypt; 4 College of Animal Science and Technology, Southwest University, Chongqing 400715, China; weiwu2019@outlook.com

**Keywords:** by-products, liver, peptides, bioactivity, taste, taste transduction

## Abstract

The liver, a by-product of livestock processing, is abundant in protein and serves as an excellent precursor for bioactive and taste-active peptides. However, comprehensive reviews on its valorization remain limited. This review summarizes recent advances in utilizing liver as a source of bioactive peptides (BPs) and taste-active peptides (TAPs). It systematically outlines the composition of liver and preparation methods of liver-derived peptides, discusses the diverse bioactivities of peptides from livestock liver and its taste characteristics, and also examines current challenges along with future perspectives. Liver-derived peptides possess various bioactivities, such as antioxidant, anti-inflammatory, antihypertensive, and metabolism-regulatory activities. Meanwhile, they can elicit the umami, sweet, and bitter taste primarily through different taste transduction receptors. The bioactive and taste properties of peptides are closely related to molecular weight, hydrophobicity, and composition of peptides. Furthermore, ultrasound treatment and the Maillard reaction are effective methods for improving the taste characteristics of liver-derived peptides. However, current research on liver-derived peptides remains limited by inefficient screening and prediction of BPs, low bioavailability, and a narrow focus on TAPs. Overall, this review provides a theoretical reference for high-value utilization of liver-derived peptides from livestock by-products.

## 1. Introduction

The global meat industry produces approximately 345 million metric tons of meat annually, while simultaneously generating around 155 million metric tons of by-products from the slaughter and processing of these animals [1]. The liver is one of the largest visceral organs in livestock and by-products, which accounts for 2–3% of body weight and makes up a substantial portion of slaughter by-products [2]. Acceptance of liver as a human food ingredient in Western markets is relatively low, largely due to consumer preferences against its sensory characteristics and associated health concerns. This mismatch between post-slaughter liver supply and demand for direct human consumption explains why a portion of livestock liver is redirected to pet food and animal feed production [3]. Nevertheless, utilization potential of livestock liver remains largely unrealized. In line with global efforts toward sustainable development and agricultural resource recycling, enhancing the value of meat by-products through environmentally friendly processing technologies has emerged as a key research priority in food science and sustainable agriculture. Accordingly, converting livestock liver into BPs and TAPs represents a promising strategy that not only offers greater economic benefits but also aligns more closely with sustainable development goals.

The liver is rich in protein and other nutrients, including vitamins A and B, minerals (e.g., iron, selenium, zinc) and various bioactive components [4]. Hydrolysis of liver proteins enables the generation of low-molecular-weight peptides with diverse bioactivities and taste-active properties, thereby facilitating high-value utilization [5]. Enzymatic hydrolysis and microbial fermentation are currently the primary methods for producing liver-derived peptides. Enzymatic hydrolysis exposes functional sequences by recognizing and cleaving specific peptide bonds, thereby enhancing bioactivity. Among proteases, Alcalase displays broad substrate specificity and preferentially cleaves peptide bonds adjacent to hydrophobic residues, yielding a high degree of hydrolysis (DH) in a short time [6]. By contrast, fermentation relies on proteases produced by microorganisms to achieve enzymatic hydrolysis. This method can increase the yield of umami and sweet peptides while reducing bitter peptide content, resulting in natural flavor enhancement, making it a suitable technology for preparing TAPs [7]. Furthermore, ultrasound-assisted technology has been demonstrated to modulate the physicochemical properties of liver-derived peptides, showing potential for enhancing the bioactive and taste-active characteristics [8].

In recent years, a growing body of research has demonstrated that liver-derived BPs from livestock possess diverse biological functions. These include antioxidant [9], antihypertensive [10], anti-inflammatory, anti-anemia [11], metabolism-promoting [6], antimicrobial [12], and gut health-promoting activities [13]. These peptides exhibit potential to be developed into functional foods. Simultaneously, liver-derived peptides are rich in umami- and sweet-tasting free amino acids, endowing them with prominent application value in food for sugar/salt reduction, flavor enhancement, and sensory quality improvement. Moreover, as precursors in the Maillard reaction, these TAPs can generate characteristic aroma compounds during thermal processing, further optimizing the overall sensory profile of products [14]. The free amino acids in TAPs also form an important basis for their taste characteristics [15]. Although existing studies have reported the diverse bioactivities of liver-derived peptides and have preliminarily explored their potential to improve sensory properties, a comprehensive review addressing their dual roles in both bioactivity and taste aspects remains lacking. Moreover, recent studies frequently omit detailed descriptions of preparation methods and their advantages, the structure–activity relationships of peptides, and the mechanisms underlying taste perception. To address these gaps, this review summarizes the nutritional composition of livestock liver, compares different preparation processes, and elucidates the associated bioactivities, taste-related properties, and underlying mechanisms, thereby providing a theoretical basis for the high value-added utilization of livestock liver.

## 2. Nutritional Composition of Liver

As a vital metabolic organ in animals, the liver supports a wide range of physiological functions through the coordinated distribution and synergistic interaction of its nutritional components. The liver exhibits vigorous metabolic and detoxification activities, which are largely dependent on various proteins involved in metabolism, nutrient storage and immune regulation. Although the nutritional composition of the liver varies across species, it is generally abundant in essential amino acids such as leucine and glutamic acid, making it a valuable source of high-quality protein [16]. This nutritional variability is evident in livestock livers.

The protein contents of livers from common livestock and poultry are shown in Table 1. After processing and evisceration, the protein proportion of chicken liver can exceed 90% [17]. In contrast, goose liver contains abundant lipids, resulting in the lowest relative protein proportion among all tested liver samples.

In addition to macronutrients, animal liver is a rich source of vitamins and minerals with notable health benefits. It is particularly high in vitamin A, which is essential for vision and epithelial integrity, reaching up to 77,400 μg/100 g in pig liver [4,21]. Liver also provides a comprehensive profile of B vitamins, including B12 (critical for red blood cell formation and nerve function), folate (involved in DNA synthesis and fetal development), and B2 and B3 (key in energy metabolism) [22]. Regarding minerals, liver contains highly bioavailable heme iron, making it ideal for preventing iron deficiency anemia; zinc, which supports immune function and wound healing; selenium, a component of antioxidant enzymes that protect against oxidative damage; and copper, involved in iron metabolism and connective tissue formation [23]. Concerning bioactive components, liver is rich in coenzyme Q10, which exhibits free radical scavenging activity; choline, which contributes to brain development and liver health; heparin, which has shown protective and reparative effects in cardiovascular diseases [24]; and glutathione peroxidase, which aids in scavenging free radicals [25].

## 3. Main Extraction Methods of Peptides from Liver

The primary extraction methods for liver-derived BPs currently include enzymatic hydrolysis, microbial fermentation, and ultrasound-assisted enzymatic hydrolysis. The extraction of peptides involves an initial pretreatment procedure. This step typically includes washing the liver tissue to remove fat and connective tissue, followed by homogenization to disrupt cellular structures and release intracellular proteins and peptides. Homogenization is usually performed at low temperatures to minimize protease activity and prevent peptide degradation. Subsequently, centrifugation is applied to remove cellular debris and undissolved tissue. Alternatively, some studies employ a pretreatment method involving freeze-drying the liver for 72 h, followed by grinding into a powder [12]. Following these pretreatment procedures, various methods can be employed for protein extraction, such as enzymatic hydrolysis, fermentation, and physicochemical methods. Notably, physicochemical methods cannot be used alone and must be combined with biochemical treatments, and all extraction strategies are summarized in Table 2. Among these, enzymatic hydrolysis and fermentation are more commonly used due to their mild reaction conditions, reduced energy consumption, and effectiveness in releasing BPs [26,27].

### 3.1. Enzymatic Hydrolysis

Enzymatic hydrolysis is a common method for extracting proteins from livestock liver. Its core principle involves the binding of specific proteases to catalytic sites on proteins under optimal pH and temperature conditions for enzyme activity. This binding leads to the cleavage of chemical bonds within the proteins, resulting in the degradation of high-molecular-weight proteins into small peptides, increased protein solubility, and ultimately, the extraction of target peptides. Following hydrolysis, the enzymes are typically inactivated by heating at an elevated temperature for a suitable duration (e.g., 90 °C for 10 min), after which the supernatant is obtained by centrifugation.

A variety of proteases are used for the enzymatic hydrolysis of livestock liver. Conventional exogenous proteases include papain, pepsin, Alcalase, trypsin, Neutrase, bromelain, and Flavourzyme. As indicated in Table 2, most studies report that Alcalase possesses excellent hydrolytic capacity, while recent studies have demonstrated that papain effectively hydrolyzes meat proteins abundant in Gly and Hyp [12]. In addition to conventional enzyme addition, some studies employ autolysis, where hydrolysis is primarily catalyzed by endogenous proteases naturally present in the liver itself [42].

Table 2 summarizes the hydrolytic capabilities of different enzymes toward livers from various animal sources. DH directly determines the molecular weight distribution, amino acid composition, and spatial conformational features (e.g., hydrophobicity) of the peptides in the protein hydrolysate. Specifically, as DH increases, proteins are progressively degraded into smaller peptides and free amino acids. This process exposes functional and taste-active peptides that were originally embedded within the tertiary structure of the proteins, thereby profoundly influencing various bioactivities, including antioxidant, antihypertensive, and anti-inflammatory effects [43]. The differences in cleavage sites among proteases lead to variations in DH, which ultimately manifest as significant differences in bioactivity.

Wang, Xing, Cai, Toldrá, Hao and Zhang [30] and Miao, Xing, Wang, Ju, Cao and Zhang [6] reported that porcine liver was hydrolyzed with Alcalase and pepsin for 5 h, respectively. The results revealed that the proportion of peptides with molecular weight (MW) < 3 kDa was 63% and 58% in these two hydrolysates, respectively. Alcalase-treated hydrolysate exhibited the highest peptide content (51.55 mg/mL) and the optimal inhibitory activity against nitric oxide (NO) production and albumin denaturation, with inhibition rates of 25.40% and 28.03%, respectively. In contrast, pepsin-treated hydrolysate had the lowest peptide content (7.33 mg/mL), with NO inhibition and albumin denaturation inhibition rates of only 9.35% and 11.13%, respectively [6]. Alcalase can hydrolyze liver proteins to produce bioactive peptides with potent bioactivities, which is determined by its catalytic characteristics. Alcalase can hydrolyze peptide bonds near hydrophobic amino acid residues, leading to rapid release of hydrophobic residues (e.g., Tyr, Trp, Phe) with free radical scavenging ability [44]. This enhanced the proton/electron-donating potential and lipophilicity of BPs, thereby improving their antioxidant capacity. Furthermore, short peptide fragments (e.g., di- and tripeptides) with aromatic C-terminal and hydrophobic N-terminal characteristics, which are exposed by hydrolysis with Alcalase, can exert antihypertensive effects by inhibiting angiotensin-converting enzyme (ACE) [45]. More detailed structure–activity relationships are discussed in Section 4.

It is noteworthy that the relationship between DH and bioactivity is not strictly positive. Moderate hydrolysis is key to enhancing bioactivity, whereas excessive hydrolysis can diminish it. For instance, Duan, Yang, Deng, Zhang, Ma, He, Zhu and Zhang [8] confirmed that the antioxidant components in bovine liver peptides are primarily concentrated in the 500–1000 Da fraction. Moderate hydrolysis can increase the proportion of peptides within this range of MW, thereby raising the relative content of antioxidant peptides. However, excessive hydrolysis led to the degradation of peptides to MW below 500 Da, which in turn resulted in a diminished antioxidant capacity of the products. Furthermore, ultrasound-assisted treatment significantly increased the content of the 500–1000 Da peptide fraction, leading to an improvement by 47.82–89.94% in the reducing power of the product compared to the control group.

Enzymatic hydrolysis is also employed in the preparation of TAPs. In terms of taste attributes, proteases such as Protamex, pepsin, papain, and bromelain tend to generate low-MW peptides (<3 kDa) with hydrophobic amino acids (e.g., Tyr, Trp) at the C-terminus. These peptides can strongly bind to bitter taste receptors. Consequently, during enzymatic hydrolysis, alongside the release of umami- and sweet-tasting free amino acids, bitter peptides are simultaneously formed and accumulated [45]. For example, López-Martínez et al. [46] reported that after hydrolyzing porcine liver with Alcalase for 30 min, the content of umami free amino acids increased from 2.869 to 6.487 mg amino acid/g liver, sweet amino acids from 5.840 to 21.068, and bitter-sweet amino acids from 2.955 to 16.390, while bitter amino acids increased from 2.563 to 22.627. However, the use of other enzymes may yield hydrolysates with more desirable taste profiles. A recent study on pig liver has revealed that sequential hydrolysis using Alcalase and Flavourzyme yields a product with a taste profile comparable to that obtained via single-enzyme hydrolysis, yet with significantly higher taste intensity [47]. It has been shown that Protease A is the optimal protease for producing hydrolysates with low bitterness and high umami [48]. Therefore, the use of Protease A on livestock liver might yield TAPs with more suitable characteristics. However, research specifically on the use of Protease A for hydrolyzing livestock liver to produce TAPs is still lacking.

### 3.2. Microbial Fermentation

The preparation of peptides via microbial fermentation relies on proteolytic enzymes synthesized and secreted by microorganisms to catalyze the degradation of proteins into small amino acid chains. This process can increase the yield and alter the composition of bioactive fractions, as well as produce abundant characteristic flavor compounds. Compared to conventional enzymatic hydrolysis, fermentation offers distinct advantages: enzymatic hydrolysis often yields bitter peptides, whereas bioactive peptides produced through microbial fermentation are characterized by the lower production profiles and more palatable flavor profiles [38]. In fermentation processes, bacterial species, such as *Lactobacillus helveticus*, *Lactobacillus delbrueckii* ssp. bulgaricus, *Lactococcus lactis* ssp. diacetylactis, *L. lactis* ssp. *Cremoris* and *Streptococcus salivarius* ssp. Thermophilus are commonly employed. The selection of specific bacterial species and strains plays a crucial role in the production of bioactive peptides [49].

As listed in Table 2, Fan, Han, Sun, Zhang, Tu, Du and Pan [38] inoculated Bacillus subtilis BNCC109047 into duck liver homogenate and obtained unique antioxidant peptides via this approach. Similarly, chicken liver hydrolysate produced using lactic acid bacterium *Pediococcus acidilactici* NCIM5368 demonstrated notable antioxidant capacity [35]. In another study, fermentation of chicken liver with *P. acidilactici* NCIM5368 generated bioactive peptides, which are responsible for recovering hemoglobin levels and significantly enhanced serum antioxidant capacity in mice. According to the study by Chakka, Ramanatikara, Zituji, Pedda and Narayan [11], oral administration of fermented chicken liver hydrolysate (FCLH) restored hemoglobin content in mice to 80% to 95% above the pre-anemia induction levels. Furthermore, the DPPH radical scavenging rate in the FCLH group ranged from 68% to 75%.

Generally, enzymatic hydrolysis achieves a higher degree of hydrolysis (DH) than microbial fermentation primarily due to the ability of enzymes to recognize and cleave specific chemical bonds in the substrate with high specificity. In contrast, microbial fermentation involves a more complex and dynamic biological process, which often results in lower recovery efficiency of target peptides [45]. However, as mentioned previously, DH does not strictly correlate with the strength of bioactivity. Indeed, both enzymatic hydrolysis and fermentation exhibit their own distinct advantages. For example, Yu, Hsu, Chang and Tan [37] found that liver hydrolysate prepared by fermentation with *Monascus purpureus* reached its highest DPPH radical scavenging activity (63%) at 12 h of fermentation. This activity was significantly superior to that of hydrolysates produced by enzymatic hydrolysis with Alcalase (42%), papain (37%), or pepsin (55%). In a comparative study, Chakka, Elias, Jini, Sakhare and Bhaskar [35] hydrolyzed chicken liver using *Pediococcus acidilactici* NCIM5368 and Alcalase. The DH values were 14.3% and 26.12%, respectively. While protein hydrolysate showed higher total antioxidant activity, the fermented product exhibited greater scavenging capacity against superoxide anions, DPPH radicals, and ABTS radicals. Regarding antimicrobial effects, FCLH exhibited strong antibacterial activity against *Listeria monocytogenes* (30 mm inhibition zone) and *Bacillus cereus* (28 mm), with moderate inhibition against *Mycoplasma luteum* and *Enterobacter coli* (both 18 mm). In contrast, the enzymatic chicken liver hydrolysate (ECLH) showed only moderate activity against *Micrococcus luteus* (12 mm) and no inhibition against the other tested organisms. Thus, ECLH and FCLH displayed different strengths in terms of antioxidant and antimicrobial activities. This differential advantage profile between ECLH and FCLH indicated that the specific exogenous enzyme or microbial species selected for enzymatic hydrolysis or fermentation directly determines the efficacy and predominant functional advantages of each method.

In terms of taste-active characteristics, products from fermentation generally exhibit superior sensory quality. While enzymatic hydrolysates tend to exhibit bitter taste, fermentation can effectively mitigate this unpleasant flavor attribute. For instance, Wang, Lan, Yang, Yang, Ma, Cheng, Xia, Xu, Wang and Zou [41] found that the contents of umami free amino acids were all elevated after fermentation. Compared with the unfermented chicken liver group, the levels of umami free amino acids Glu and Asp in the conventionally fermented chicken liver group increased by 14.6% and 32.3%, respectively. Meanwhile, the bitter free amino acids Val and Leu decreased by 37.9% and 41.4%, respectively. Fermentation also facilitates the progression of the Maillard reaction in products to improve palatability and sensory properties, as detailed in Section 5.2.

### 3.3. Ultrasound-Assisted Extraction

Ultrasound-assisted enzymatic hydrolysis and microbial fermentation are common approaches in the preparation of liver-derived peptides, often applied either before or after the main hydrolysis or fermentation step. Zou et al. [50] reported treating water-soluble chicken liver protein isolates subjected to high-speed homogenization with low-frequency ultrasound. This treatment induced significant alterations in the secondary and tertiary structures of the protein as well as its thermal denaturation properties, subsequently modifying its bioactivity and taste characteristics.

Ultrasound-assisted enzymatic hydrolysis can modify the profile of liver-derived peptides and yield fractions with higher bioactivities. Zhao, He, Dang, Cao, Sun and Pan [40] treated defatted goose liver powder with 20 kHz ultrasonic cell disruptor at different power levels (0, 300, and 600 W) for 30 min, followed by pepsin hydrolysis for 2 h. The results indicated that, compared to the control, the IC_50_ of the initial hydroxyl radicals decreased by 19.23% and 11.26% in the 300 W and 600 W treatment groups, respectively, confirming that ultrasound-assisted treatment effectively enhanced the antioxidant activity of the product. In antibacterial studies, Chen et al. [51] found that Maillard reaction products derived from ultrasound-treated chicken liver protein hydrolysates carried more negative charges than those obtained from untreated chicken liver protein hydrolysates, thereby effectively inhibiting the growth of saprophytic bacteria and *Escherichia coli*. This enhancement is attributed to the cavitation effect of ultrasound, which can intensify interactions between protein molecules, expose hidden groups, or alter the secondary structure of proteins such as the water-soluble protein from chicken liver, thereby influencing the bioactivity of the resulting peptides [50]. For example, Duan, Yang, Deng, Zhang, Ma, He, Zhu and Zhang [8] observed that after treating a bovine liver alkaline protease hydrolysate with 500 W ultrasound, the proportion of peptide fraction (500–1000 Da) increased by approximately 13.8%. The underlying reasons for this effect are as follows. First, 600 W ultrasound treatment significantly altered the secondary structure of the liver protein/peptide mixture, increasing the β-sheet content (from 40.25% to 46.95%), while decreasing the α-helix (from 15.90% to 13.99%) and β-turn (from 28.95% to 24.93%) contents (*p* < 0.05). Notably, the relationship between ultrasound power and bioactivity enhancement did not show a strictly positive correlation. In the study by Zou, Shi, Chen, Xu, Jiang, Xu and Wang [50], although the 600 W group exhibited higher hydroxyl radical scavenging capacity than the control group, it was lower than that of the 300 W group.

Besides ultrasound power, hydrolysis time, substrate concentration, and enzyme activity also influence DH and bioactivity. For example, Romero-Garay, Adaile-Pérez, Montalvo-González, Martínez-Montaño and García-Magaña [39] treated samples with 560 W ultrasound for 20 min at an enzyme-to-substrate (E/S) ratio of 0.1, followed by hydrolysis for either 75 or 150 min. The group hydrolyzed for 150 min showed significantly higher DH (97.09% vs. 41.33%) and ABTS radical scavenging activity (485.34 µM TE vs. 476.35 µM TE) compared to the 75 min group. However, no significant difference was observed in Fe^2+^ chelating ability, and the FRAP value was lower in the 150 min group (69 mM TE vs. 88.95 mM TE). When ultrasound time was 20 min, hydrolysis time 150 min, and the E/S ratio 0.05, the DH (39.73%), ABTS activity (481.26 µM TE), and FRAP value (59.13 mM TE) were all significantly lower than those in the group with E/S ratio of 0.1, while Fe^2+^ chelating ability again showed no significant difference, indicating weaker overall antioxidant capacity. However, this trend of weaker antioxidant capacity at the lower E/S ratio was not observed under conditions of ultrasound treatment for 10 min and hydrolysis for 112.5 min. Overall, hydrolysis time and E/S ratio are key process parameters regulating DH and bioactivity. However, their effects on promoting the functional efficacy of hydrolysates are not always positively correlated.

Ultrasound-assisted enzymatic hydrolysis is also applied in the preparation of liver-derived TAPs. Appropriate selection of ultrasonic power, treatment time, and enzyme type can effectively improve the taste characteristics of the product. López-Martínez, Toldrá and Mora [46] found that ultrasound probe-assisted hydrolysis with Alcalase significantly increased the contents of His, Leu, and Phe (*p* < 0.05), leading to enhanced bitterness that could adversely affect the overall flavor. In contrast, treating heat-treated liver hydrolysate with Flavourzyme for 30 min nearly doubled umami intensity (increasing from 9370 to 16,497), suggesting that the enhanced umami may mask the increased bitterness. The hydrolysis method also significantly affects the release of taste-active amino acids, with sequential hydrolysis proving markedly superior to single-step hydrolysis. López-Martínez, Toldrá and Mora [47] confirmed that sequentially hydrolyzed products contained the highest levels of umami amino acids. The release of Asp, Glu, and total umami amino acids increased approximately 4-fold (from 2.885 to 12.449 mg amino acid/g liver), 3-fold (from 6.257 to 18.856 mg/g), and more than 3-fold (from 9.142 to 31.305 mg/g), respectively, compared to single-step hydrolysates. Furthermore, López-Martínez, Toldrá and Mora [47] reported that both ultrasonic bath pretreatment (11.501 mg amino acids AA/g liver) and ultrasonic probe pretreatment (11.505 mg AA/g liver) increased the total umami amino acid content in Alcalase hydrolysates compared to the group without ultrasonic treatment (9.142 mg AA/g liver). However, the intensity of ultrasound pretreatment should not be excessive, as prolonged sonication can lead to sample overheating. This thermal effect may not only cause thermal inactivation of the enzyme but also induce protein aggregation, thereby reducing protease accessibility and ultimately diminishing umami intensity [47].

Ultrasound-assisted microbial fermentation also holds potential for improving taste characteristics. For instance, Wang, Lan, Yang, Yang, Ma, Cheng, Xia, Xu, Wang and Zou [41] fermented chicken liver with *Pediococcus pentosaceus* assisted by ultrasound at power of 560 W for 30 min. Following ultrasound-assisted fermentation, the total free amino acid and hydrolyzed amino acid contents in chicken liver increased by 39.38% and 41.77%, respectively. The contents of Asp and Glu were also significantly increased after fermentation. This enhancement was attributed to ultrasound facilitating the interaction between proteases produced by *P. pentosaceus* and the protein substrate, leading to changes in amino acid content. Taken together, ultrasound-assisted technology can effectively optimize both bioactivity and taste characteristics of liver-derived peptides. However, to maximize product quality, it is essential to strictly control the relevant process parameters (e.g., ultrasound, hydrolysis and fermentation conditions), select protease types, and optimize hydrolysis strategies.

## 4. Bioactive Peptides Derived from Livestock Liver

**Figure 1:**
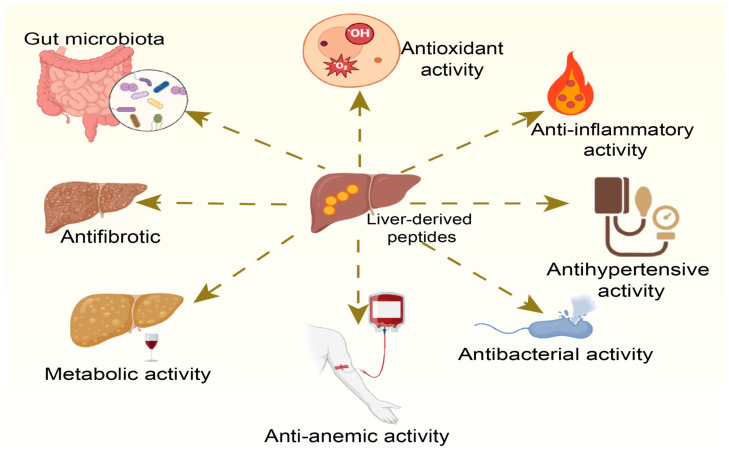
The diverse bioactivities of liver-derived peptides and their regulatory effects on cardiovascular system, liver protection, intestinal flora, and other physiological processes.

Liver-derived peptides have been reported to possess diverse bioactivities. Figure 1 integrates their primary activity types, including antioxidant, anti-inflammatory, antihypertensive, metabolism-regulating, gut microbiota-modulating, antimicrobial, and anti-anemia activities. The functional efficacy of bioactive peptides is often closely related to the amino acid composition and structural characteristics [52]. The main sources, preparation methods, bioactivities, and structural characteristics of bioactive peptides derived from liver are summarized in Table 3. Notably, current research on the bioactivity of liver-derived peptides is predominantly confined to in vitro experiments, with a scarcity of animal and clinical trials. Accordingly, most experimental data presented in this paper originate from in vitro analyses. This limitation is discussed in Section 6.

### 4.1. Antioxidant Activity

Antioxidant activity is of significant importance in both food systems and organismal metabolism. In food systems, antioxidants can inhibit the oxidative deterioration of lipids and proteins triggered by reactive oxygen species (ROS), thereby delaying food quality decline and ensuring stability and safety [56]. Excessive reactive oxygen species produced in the body under pathological or extreme stress conditions can damage biological macromolecules such as proteins and lipids, atherosclerosis, tumors and various liver diseases mediated by oxidative stress [57]. As natural antioxidants with the merits of simple structure, easy absorption and favorable stability, antioxidant peptides can effectively scavenge free radicals and inhibit lipid oxidation, thus exerting great significance for food quality maintenance and human health [58,59,60]. Peptides exhibit distinct differences in intrinsic antioxidant potential, which are mainly determined by several core factors. The amino acid composition, relative molecular weight, and preparation procedure of hydrolysates are critical factors affecting the antioxidant capacity of liver-derived peptides. Currently, a variety of methods are commonly used to assess the antioxidant capacity of peptides. These include the DPPH and ABTS radical scavenging assays, FRAP assay, ORAC assay, Fe^2+^ chelating ability measurement, ferric reducing power assay, hydroxyl radical scavenging assay, and evaluation using cellular oxidative damage models. These methods differ significantly in their reaction mechanisms, detection principles, and applicable scopes, thereby reflecting the antioxidant efficacy of peptides from different dimensions. Notably, while various methods are used to determine antioxidant activity, each can only reflect a specific dimension of antioxidant action. Consequently, results from different assays are often not entirely consistent, necessitating combined analysis for a more comprehensive understanding [61].

Currently, research on the remarkable antioxidant activity of liver-derived peptides mainly focuses on in vitro investigations. Sun, Zhou, Cao, He, Sun, Dang, Pan and Xia [32] treated duck liver with Alcalase and found that a subfraction of the resulting hydrolysate (1 mg/mL) exhibited the highest antioxidant activity among the tested groups, with DPPH radical scavenging rate of 45.55%, ABTS radical scavenging rate of 77.68%, and hydroxyl radical scavenging rate of 24.06%. Similarly, Duan, Deng, Yang, Zhang, Ma, He, Ma, Li and Li [33] reported that DPPH scavenging rates for bovine liver hydrolysates prepared with alkaline protease and papain ranged from 21.3% to 47.3% and 19.9% to 43.4%, respectively. The Fe^2+^ chelating capacity of the alkaline protease hydrolysate was 24.4% higher than that of the pepsin-treated hydrolysate. Furthermore, the ferric reducing power of the Alcalase-treated hydrolysate (5 mg/mL) was 0.517 AU (absorbance units at 700 nm), significantly higher than that of hydrolysates produced by other proteases (0.305–0.490 AU). Wang, Xing, Cai, Toldrá, Hao and Zhang [30] also confirmed that when pig liver hydrolysate produced by Alcalase reached the concentration of 2 mg/mL, the DPPH and ABTS radical scavenging rates reached 79.02% and 79.72%, respectively, demonstrating strong antioxidant potential. Di Bernardini et al. [62] also reached a similar conclusion.

Although animal experimental evidence regarding its antioxidant effects is relatively limited, relevant studies can provide more supporting data. For instance, Chen et al. [63] showed that chicken liver-derived peptides could extend the lifespan of *Caenorhabditis elegans*. Treatment with the liver-derived peptide WYR enhanced the stress resistance of the nematodes under oxidative stress, with a survival rate reaching 49.81% at 0.4 mg/mL, approximately three times that of the control group (16.82%). Moreover, Chou et al. [64] established the D-galactose-induced aging mouse model to investigate the antioxidant capacity of peptides obtained from pepsin-hydrolyzed chicken liver. Long-term intake of D-galactose triggers excessive production of reactive free radicals. The antioxidant activity of the peptides was evaluated by measuring the accumulation of malondialdehyde (MDA, a secondary lipid oxidation product) and the levels of three antioxidant enzymes, including CAT, GPx and SOD. After intragastric administration of chicken liver hydrolysates at doses of 0.05 and 0.25 g/kg, the antioxidant capacity of mouse brain, heart, liver and kidney was comparable to or even higher than that of the blank control and model groups. Doses of 0.25 and 0.5 g/kg effectively suppressed lipid oxidation in serum and liver, with oxidation levels showing no significant difference from the control group. D-galactose treatment reduced the activity of endogenous antioxidant enzymes, whereas chicken liver hydrolysates markedly elevated the activities of CAT, GPx and SOD in mouse serum and liver, restoring them to the levels observed in normal control mice.

The antioxidant mechanisms of liver-derived antioxidant peptides are complex, which are primarily associated with various factors, including amino acid composition and sequence, relative molecular weight, peptide structural features, and physiological regulatory functions. These influencing factors are elaborated as follows. First, amino acid composition and sequence serve as the core determinants of antioxidant activity of peptides. Among them, hydrophobic amino acids (Gly, Ala, Val, Trp, Phe, Ile, Leu, Pro, and Met) can enhance the lipid solubility of peptides, strengthen their interaction with free radicals, and consequently block the chain reaction of lipid peroxidation. For instance, Trp, Met, and Ala have been identified as key amino acids at the binding sites of antioxidant peptides with DPPH or ABTS radicals [32]. Aromatic amino acids (Tyr, His, Trp and Phe) can donate protons to electron-deficient free radicals to stabilize them, while maintaining their own molecular stability via resonance structures. Specifically, the imidazole ring of His enables hydrogen donation, trapping of lipid peroxyl radicals, and metal ion chelation. Cys, due to its thiol (-SH) group, can act as a radical scavenger to enhance antioxidant capacity [37]. For example, in the study by Gallego, Mora and Toldrá [10], the peptide SWGPGIP was found to possess antioxidant capacity, potentially due to the presence of the aromatic amino acid Trp and the hydrophobic amino acids Gly, Pro, and Ile. Acidic amino acids (Asp and Glu) and basic amino acids (Arg and Lys) can utilize their side-chain carbonyl and amino groups as chelators for metal ions. Furthermore, acidic amino acids, such as Glu and Asp can generate excess electrons upon interaction with free radicals, contributing to the antioxidant capacity of the peptide [33,65].

As another critical component of peptide primary structure, amino acid sequence arrangement synergistically modulates the antioxidant activity of peptides in combination with amino acid composition. For instance, peptides containing Leu or His at the N-terminal position exhibit higher antioxidant activity [65], which may promote interactions with fatty acids. Moreover, peptides exhibit prominent antioxidant activity when valine is located at the N-terminus, or when they contain Tyr, Trp, Ala, Pro, Met, Lys, Asp or Cys residues within their sequences [37]. Dipeptides composed of Tyr or Trp at the amino terminus, as well as those with His or Met at the carboxyl terminus, tend to exhibit stronger antioxidant activity [65]. Pearman, Ronander, Smith and Morris [9] reported that peptides such as FWG, MLFG, and SDPPLVFVG were selected as antioxidant peptides partly due to Gly residue at the C-terminus. Similarly, Fan, Han, Sun, Zhang, Tu, Du and Pan [38] reported detailed molecular interactions for the identified peptides.

The molecular weight can exert an important impact on the antioxidant mechanisms of liver-derived antioxidant peptides. Typically, antioxidant peptides from food sources have a low molecular mass, generally ranging from 500 to 1800 Da [32], as molecular weight affects the pathway by which bioactive peptides reach their targets. Compared to proteins or single amino acids, peptides containing 2–6 amino acid residues are more readily absorbed through the gastrointestinal barrier and enter the peripheral bloodstream [65]. Fan, Han, Sun, Zhang, Tu, Du and Pan [38] fermented duck liver with Bacillus subtilis and identified four antioxidant peptides, namely MYGAVTPVK, NWEKIR, APGIIPR, and RWWQLR. These peptides consist of six to nine amino acid residues, with molecular weight ranging from 723.45 to 981.51 Da, further corroborating the advantageous characteristics of short peptides in terms of antioxidant activity.

The structural features of peptides can also impact their antioxidant effects. Ultrasound-assisted treatment can promote antioxidant effects by modifying structural features. The specific mechanism underlying structural changes has been elaborated in Section 3.3. Collectively, these alterations in structure and chemical properties create favorable conditions for subsequent enzymatic hydrolysis and the enhancement of antioxidant activity [8].

Beyond the direct free radical-scavenging capacity of peptide-bound amino acid residues, bioactive peptides can also exert antioxidant effects by regulating intracellular antioxidant signaling pathways. For instance, Fan, Han, Sun, Zhang, Tu, Du and Pan [38] demonstrated that liver-derived peptides MYGAVTPVK, NWEKIR, APGIIPR, and RWWQLR exert antioxidant activity, which may be mediated by upregulating the mRNA expression levels of antioxidant enzymes, thereby protecting HepG2 cells from oxidative stress. At the concentration of 1 mg/mL, the peptide MYGAVTPVK significantly elevated the mRNA levels of SOD, CAT, and GSH-Px compared with the blank control group. The mRNA expression of these three antioxidant enzymes was also markedly higher than that of the oxidative damage group, reaching at least three times the expression level of the oxidative stress model group. Furthermore, Du, Chen, Wei, Zhu and Cai [36] found that LPLPFP, derived from the NADH-ubiquinone oxidoreductase chain 1 in goose liver, could inhibit ROS production and prevent oxidative stress damage in HHL-5 hepatocytes by upregulating gene expression in the Ahr-NQO1 signaling pathway.

### 4.2. Anti-Inflammatory Activity

Chronic inflammation represents a long-term response of the body’s immune system to harmful stimuli and is associated with the onset and progression of various diseases, including cardiovascular diseases, metabolic disorders, and even cancer [66]. Currently, side effects such as gastrointestinal disorders and an increased risk of cardiovascular disease are associated with anti-inflammatory drugs [67]. Therefore, development of safe and effective natural anti-inflammatory peptides has become a key research focus. Inhibitory rates of nitric oxide release and albumin denaturation are common in vitro indicators for evaluating anti-inflammatory activity, which reflect the level of pro-inflammatory mediators and the degree of inflammation-induced protein denaturation, respectively [6]. Meanwhile, pro-inflammatory factors including TNF-α, IL-6 and NF-κB can directly reveal the regulatory effect of peptides on inflammatory signaling pathways. Testing protocols vary across studies. Miao, Xing, Wang, Ju, Cao and Zhang [6] only determined the inhibitory activities against albumin denaturation and NO production, whereas Fan, Zhang, Sun, Zhou, Xia, Du, Wu and Pan [53] combined measurements of NO and multiple pro-inflammatory factors to assess the anti-inflammatory capacity of liver-derived peptides more comprehensively and accurately.

Liver-derived peptides possess significant anti-inflammatory activity. At the in vitro research level, liver-derived peptides exhibit remarkable anti-inflammatory activity. The anti-inflammatory effects of peptides obtained from different liver sources and via different preparation methods demonstrate clear concentration dependence and structure correlation. Miao, Xing, Wang, Ju, Cao and Zhang [6] found that porcine liver protein hydrolysate at 1 mg/mL exhibited 23.28% inhibition rate of albumin denaturation and at 5 mg/mL showed 25.10% inhibition rate of NO release. Peptides such as LFWFR, FFVFPR, DSFFPR, NSFFPR, NPLLFR, NFPLLK, and DFPLLK from this hydrolysate were identified as capable of inhibiting the secretion of inflammatory cytokines. Furthermore, Laidi, Yangying, Changyu, Daodong and Jun [54] also proved that three smaller molecular weight novel peptides screened from duck liver could inhibit the release of TNF-α and IL-6 in LPS-induced RAW264.7 cells. Among them, the peptide VIESPPEI at a concentration of 100 μg/mL achieved 42.48% inhibition rate against TNF-α release. IDVSPDSPDHY exhibited the strongest ability to inhibit IL-6 release, with an inhibition rate of 27.04% at 25 μg/mL.

Amino acid composition, terminal structure, and the regulation of inflammation-related signaling pathways are key to the function of liver-derived anti-inflammatory peptides. Laidi, Yangying, Changyu, Daodong and Jun [54] indicated that anti-inflammatory peptides are rich in hydrophobic amino acids and positively charged amino acids, especially at the N- or C-terminus. Consistent with the action mechanism of antioxidant peptides, hydrophobic amino acids facilitate peptides to preferentially penetrate cell membranes and interact with cytoplasmic proteins to exert anti-inflammatory effects. Phe exhibits low susceptibility to degradation by intestinal proteases and peptidases, which helps maintain the structural stability of peptides. Positively charged regions of cationic amino acids may bind to chemokines and their corresponding receptors to modulate immune responses [68]. In the study conducted by Laidi, Yangying, Changyu, Daodong and Jun [54], the peptide IDVSPDSPDHY contains a hydrophobic tyrosine residue at its C-terminus, while LVYPFPGPI and VIESPPEI possess hydrophobic leucine and valine residues at their respective N-termini, and all three peptides carry isoleucine residues within their sequences. In their study, the peptide IDVSPDSPDHY contains a hydrophobic Tyr residue at the C-terminus, while LVYPFPGPI and VIESPPEI contain hydrophobic Leu and Val residues at their N-termini, respectively. All three peptides contain Ile residues. Furthermore, adding two or four Phe residues can increase peptide hydrophobicity; the peptides IDVSPDSPDHY and VIESPPEI obtained in that study both contain two Phe. Beyond the role of amino acids, regulating inflammation-related genes and signaling pathways is also a core mechanism for anti-inflammatory peptides. Fan, Zhang, Sun, Zhou, Xia, Du, Wu and Pan [53] found that IDVSPDSPDHY, LVYPFPGPI, and VIESPPEI inhibited mRNA expression of TNF-α in RAW264.7 cells by 50.50%, 59.28%, and 44.60%, respectively. IDVSPDSPDHY at 25 μg/mL achieved 16.37% inhibition of IL-6 mRNA. IDVSPDSPDHY and LVYPFPGPI at 50 μg/mL showed the strongest inhibition of COX-2 mRNA, with inhibition rates of 79.41% and 84.03%, respectively. Moreover, both P3 and P10 reduced NF-κB mRNA expression in a dose-dependent manner, reaching inhibition rates of 47.64% and 30.93% at 100 μg/mL, respectively. It was concluded that liver peptides exerted their effects by regulating NF-κB signaling pathway, potentially through inhibiting IκBα protein phosphorylation and NF-κB p65 nuclear translocation.

### 4.3. Antihypertensive Activity

Hypertension, as a common chronic disease, is directly or indirectly associated with risks of cardiovascular diseases, stroke, angina, renal failure, and diabetes [69]. Peptides derived from liver can be evaluated for potential antihypertensive activity through their inhibitory effects on the ACE-I and ECE-I enzymes. ACE-I catalyzes the conversion of angiotensin I to the potent vasoconstrictor angiotensin II and can degrade the vasodilatory bradykinin; therefore, ACE-I inhibitors serve as an alternative approach for treating hypertension. Furthermore, the multifaceted functions of liver-derived bioactive peptides, such as regulating lipid metabolism, anti-inflammatory, and antioxidant activities, may work in concert with ACE-I inhibitory activity to protect cardiovascular health.

In current studies, the antihypertensive activity of liver-derived peptides is mainly predicted via computer simulation. López-Pedrouso, Lorenzo, Bou, Vazquez, Valcarcel, Toldrà and Franco [42] found that porcine liver hydrolysate at pH 7.5 could achieve an ACE inhibition rate of 65.42%. Their study employed the BIOPEP UWM database for the virtual prediction of bioactive peptides among liver-derived sequences, identifying YGLAAAVFTK, INYGGEIPK, GILAADESTGSIAKR, VAFTGSTQVGK, AGAGSATLSMAYAGAR, VGDKVLLPEYGGTK, AYQDQKPGTSGLR, IIPPGSGIIHQVNLEYLAR, and AAWAFSR as peptides with high ACE inhibitory activity. Similarly, Fan, Zhang, Sun, Zhou, Xia, Du, Wu and Pan [53], using LC-MS/MS, identified ten peptides from duck liver, namely DLTGIPPAP, ELKPTPEGDL, IDVSPDSPDHY, IYVDAVINH, LDSNLDLKF, LGEHNIDV, LVYPFPGPI, QTNLVPYPR, SLVYPFPGPIPN, and VIESPPEI, which were also demonstrated by machine learning to possess ACE inhibitory activity. Correspondingly, bovine liver hydrolysates PF (pepsin followed by Flavourzyme), PUS1F (ultrasound-assisted pepsin for 1 h followed by Flavourzyme), and PUS2F showed ACE-I inhibition rates of approximately 80% at a concentration of 10 mg/mL [10].

Structurally, ACE inhibitory activity of peptide often depends on the amino acids at the termini of the peptide sequence. The N-terminus frequently features hydrophobic amino acids with aliphatic chains (e.g., Gly, Ile, Leu, Val), while the C-terminus often features amino acids with ring structures or aromatic rings (e.g., Pro, Tyr, Trp) [10]. For instance, peptide IDVSPDSPDHY serves as an example of this pattern. However, despite known compositional trends in antihypertensive peptides, the specific structure–activity relationship governing the hypotensive activity of liver-derived peptides has yet to be established, warranting further mechanistic investigation.

### 4.4. Metabolic Modulatory Activity

Bioactive peptides show significant potential in the regulation of lipid metabolism for prevention and treatment purposes. Abnormal lipid metabolism can lead to fatty liver disease and may contribute to insulin resistance, thereby affecting systemic glucose metabolism [70]. Liver-derived functional peptides can regulate lipid metabolism through multiple pathways, improving pathological conditions related to glucose and lipid metabolism disorders. In in vitro cell assays, Miao, Xing, Wang, Ju, Cao and Zhang [6] found that PLPHs, represented by bioactive peptides such as LFWFR, FFVFPR, DSFFPR, NSFFPR, NPLLFR, NFPLLK, and DFPLLK, reduced lipid accumulation in HepG-2 cells. Compared to the palmitic acid group, supplementation with PLPHs (20 μg/mL) decreased TC and TG levels by 49.63% and 41.51%, respectively. This regulatory effect is closely related to core pathways of cellular lipogenesis and metabolism, such as downregulating the expression of certain mRNAs associated with adipogenesis. Furthermore, treatment with 20 μg/mL PLPHs inhibited the relative mRNA expression levels of FAS, ACC, and CD36 by 50.33%, 16.39%, and 48.17%, respectively, and PPAR-γ mRNA expression also decreased by 24.83%. In animal model studies, Yang et al. [71] reported a hamster-based study demonstrating that supplementation with chicken liver hydrolysate (CLH) at doses of 200 mg/kg body weight and above under high-fat feeding significantly elevated serum high-density lipoprotein cholesterol (HDL-C) levels while markedly reducing low-density lipoprotein cholesterol (LDL-C) levels. This improved the serum cholesterol profile, with the LDL-C/HDL-C ratio decreasing by up to 28.2% relative to the high-fat diet group, which reflected the regulatory effect of liver-derived peptides on lipid homeostasis in high-fat-diet-fed hamsters. In addition, Lu, Zhou, Lou, Gao, Li, Gao, Han, Liu, Xu, Qi, Chen and Zhou [13] found that the liver-derived bioactive peptide KITGLGVK can alleviate disorders in short-chain fatty acid (SCFA) production, bile acid (BA) metabolism, and energy metabolism via the gut–liver axis, thereby regulating cholesterol balance and increasing ATP generation.

In addition to regulating lipid metabolism, liver-derived peptides also demonstrate a significant promoting effect on alcohol metabolism. Lin et al. [72] confirmed that chicken liver-derived peptides can accelerate alcohol clearance by enhancing the activities of alcohol dehydrogenase and acetaldehyde dehydrogenase. Blood alcohol levels in groups treated with BCM95 (a curcumin-based product) and GBHP01 (a chicken-liver hydrolysate-based formula capsule) were 74.16% and 79.75% lower than the control group, respectively. Concurrently, GBHP01 supplementation reduced cytochrome P450 2E1 protein expression in binge-drinking rats. The study also showed increased hepatic antioxidant capacity, a possible reduction in these abnormal pathological changes and an increase in the number of intact polygonal hepatocytes. Furthermore, improvements were observed in reduced intestinal permeability (decreased ZO-1 protein) and upregulated serum endotoxin levels, along with significant downregulation of inflammatory cytokine levels. Compared with the alcohol group, IL-1β decreased by 38.92%, IL-6 by 25.16%, and TNF-α by 41.25%, respectively. Wang, Xing, Cai, Toldrá, Hao and Zhang [30] reported that the liver-derived peptide NTLPHPTAP from porcine liver could enhance the activities of ADH and ALDH via hydrogen bonds and hydrophobic interactions. The aforementioned research demonstrates that liver-derived peptides can effectively promote alcohol metabolism and alleviate alcohol-induced toxic damage. In the aspects of lipid metabolism regulation, alcohol metabolism promotion, and antioxidant function, liver-derived metabolism-regulating peptides exhibit significant hepatoprotective effects, holding application potential as liver-protective bioactive peptides.

### 4.5. Antimicrobial Activity

Liver-derived peptides can also exhibit certain antimicrobial activity, with advantages such as a broad spectrum and low cytotoxicity. These advantages endow them potential as alternatives to preservatives and possibly as substitutes for synthetic antibiotics [45]. Duck LEAP-2 peptide has been shown to possess antimicrobial activity. Hong et al. [73] conducted bacteriostatic experiments using liver-derived LEAP-2 peptide at different concentrations. The results indicated that this peptide has significant bacteriostatic activity; at suitable concentrations, the cell survival rate was close to 0, indicating possible complete eradication. Furthermore, LEAP-2 containing disulfide bonds showed significantly higher antimicrobial activity than linear LEAP-2. Specifically, disulfide-bonded LEAP-2 at a concentration of 50 μg/mL efficiently eradicated Staphylococcus aureus and *Listeria monocytogenes*. Against *Escherichia coli*, linear LEAP-2 at 200 μg/mL achieved an inhibitory rate of 60%, while disulfide-bonded LEAP-2 at 50 μg/mL achieved an inhibitory rate exceeding 90%. Both peptides at 200 μg/mL showed inhibition rates exceeding 70% against *Salmonella choleraesuis*. Against *Salmonella typhimurium*, linear LEAP-2 had a killing rate of 50%, while disulfide-bonded LEAP-2 at 200 μg/mL reached a proliferation inhibition rate of 90%. Against *Salmonella enteritidis*, disulfide-bonded LEAP-2 had an inhibition rate of 60%, while linear LEAP-2 showed no significant inhibitory effect. This study confirmed the good antimicrobial capacity of liver-derived peptides. Borrajo, López-Pedrouso, Franco, Pateiro and Lorenzo [29] reported that 30 kDa membrane-separated fraction (close to the whole component) of porcine liver hydrolysate treated with Flavourzyme showed an increasing inhibition rate against Brochothrix thermosphacta over time: 83.3% at 4 h and 116.7% at 10 h. The 10 kDa membrane-separated fraction showed the same trend, with inhibition rates of 66.7% at 4 h and 100% at 10 h. Juknienė, Zaborskienė, Jankauskienė and Mačionienė [12] also confirmed that hydrolysate from bovine liver treated with papain for 24 h exhibited antimicrobial efficacy (17.5 mm against *L. monocytogenes*, 10.0 mm against *S. enterica* subsp. enterica serovar Typhimurium). This study revealed that, within certain conditions, the antimicrobial activity of bovine liver peptides against *L. monocytogenes* was inversely proportional to the contents of Glu, His, and Lys.

The primary mechanisms of antimicrobial peptides (AMPs) are divided into membrane targets and intracellular targets. Most AMPs act on the cell membrane as their target, achieving antimicrobial effects by binding to negatively charged residues on the membrane or relying on hydrophobic interactions to damage the membrane. For example, LEAP-2, when the pH was lower than its pI (9.11), can bind to negatively charged residues on the bacterial cell wall or membrane. Subsequently, it disrupted the cell envelope by pore formation, leading to leakage of cellular contents and cell death [73]. Gram-negative bacteria have an outer membrane in their cell wall structure, so the inhibitory effect of AMPs was generally higher against Gram-positive bacteria than Gram-negative bacteria, leading to leakage of cellular contents and cell death [73]. For instance, in the study by Borrajo, López-Pedrouso, Franco, Pateiro and Lorenzo [29], porcine liver hydrolysates did not show growth-inhibitory effects against Gram-negative bacteria. On the other hand, a small number of AMPs specifically bind to intracellular targets, inhibiting the synthesis of cellular macromolecules (such as nucleic acids, proteins) to exert antimicrobial effects. Additionally, AMPs may enter mitochondria, affecting the activity of mitochondrial enzymes, causing metabolic disorders, and ultimately leading to cell death [2]. Therefore, using liver as a precursor to prepare antimicrobial peptides can provide important raw material support for the development of natural antimicrobial agents. However, it has been shown that the antimicrobial effect of liver-derived AMPs against Gram-negative bacteria is relatively limited.

### 4.6. Other Bioactivities

In addition to the bioactivities mentioned above, bioactive peptides derived from liver also demonstrate potential in modulating the gut microbiota and exerting anti-anemia effects. Research indicates a close connection between gut microbiota and liver health. As the first organ to encounter nutrients absorbed from the intestine and microbial metabolites, disruption of gut microbiota caused by dietary or environmental changes is a risk factor for liver diseases, associated with both alcohol-related and non-alcohol-related liver diseases [74]. Therefore, the effect of liver-derived peptides on gut microbiota may have beneficial implications for liver health. Lu, Zhou, Lou, Gao, Li, Gao, Han, Liu, Xu, Qi, Chen and Zhou [13] found that the porcine liver bioactive peptide KITGLGVKKIT effectively reversed abnormal levels of liver function markers (AST, ALT, TC, TG, TBA, and TBIL) in rats via the gut–liver axis, improved intestinal barrier damage (normalizing levels of LPS, DAO, DLA, occludin, and claudin-1), significantly reduced the abundance of Bacteroides and *Sphingomonas*, while restoring *Lactobacillus*, *Pediococcus*, and *Faecalibacterium*, thereby normalizing the levels of key intestinal metabolites (SCFAs, bile acids, energy metabolites, microbial metabolites). Regarding anti-anemia activity, Chakka, Ramanatikara, Zituji, Pedda and Narayan [11] demonstrated that protease-hydrolyzed chicken liver hydrolysate could restore hemoglobin content to over 95% of the original level in Swiss-albino female mice with anemia induced by cyclophosphamide (100 mg/kg), indicating its potential to alleviate iron deficiency anemia.

Overall, numerous existing studies have verified the diverse bioactive potential of liver-derived hydrolysates. Liver-derived bioactive peptides exert antioxidant and anti-inflammatory effects as their fundamental biological functions. Beyond that, they exhibit the potential to protect cardiovascular health by modulating lipid metabolism, mitigating abnormal lipid deposition on vascular walls, and exerting antihypertensive effects. Furthermore, these peptides also display promising potential for regulating alcohol metabolism and exhibit notable antimicrobial activity. In terms of structure–activity relationships, biological activity is not determined solely by relative molecular weight or a single type of amino acid. The optimal functional performance of peptides is generally governed by the synergistic effects of multiple factors, including specific amino acid compositions (such as the presence of hydrophobic amino acids, aromatic amino acids and positively charged amino acid residues), low molecular weight (typically less than 1500 Da), amino acid sequence arrangement (e.g., amino acids at the N-terminus and C-terminus), peptide spatial conformation, and the regulation of cellular signaling pathways [75].

According to the foregoing content, metabolic modulatory activity is supported by cell experiments and a small number of animal studies; anti-inflammatory activity has been extensively investigated in vitro but lacks animal experimental validation; and most studies on ACE inhibitory activity rely on computer simulation. Therefore, existing evidence is insufficient to verify their efficacy in humans, and more animal or clinical trials are required to validate the above-mentioned bioactivities. In terms of antioxidant activity, abundant in vitro studies have indicated its potential as a substitute for synthetic antioxidants. Nevertheless, supplementary animal and clinical investigations are necessary if liver-derived peptides are to be developed into functional foods that exert beneficial effects in the human body. Regarding antimicrobial activity, relevant research mainly focuses on in vitro performance given its application prospect as an alternative preservative in the food industry, yet no studies have confirmed its activity in diverse food matrices. Detailed limitations and corresponding countermeasures are presented in Section 6: Future Research Directions and Challenges.

## 5. TAPs Derived from Livestock Liver

Taste-active peptides are short peptides or oligopeptides related to taste perception, with MW below 3000 Da. They can elicit taste sensations themselves and are typically categorized by their taste characteristics, such as sweet, umami, bitter, sour, and salty taste [76]. The amino acid profile of liver proteins confers a distinct advantage for their use as a source of TAPs. López-Martínez, Toldrá and Mora [15] found that porcine liver had a significantly higher total concentration of all taste-related free amino acids (11.497 mg/g organ) than brain, kidney and lung (*p* < 0.05). Within these free amino acids, the total content of sweet-tasting free amino acids (esp. Ser, Gly, Gln, Thr and Ala) reached 3.870 mg amino acids/g organ, which was also markedly higher than that in the other three organs. Moreover, the content of umami free amino acids (esp. Asp and Glu: 2.457 mg/g organ) in porcine liver exceeded the other three organs as well.

### 5.1. Taste Activity of Liver-Derived Peptides

The taste of liver-derived peptides is primarily evaluated by the taste activity value (TAV). It is calculated as the ratio of the actual concentration of a compound (typically a free taste-active amino acid) to its sensory threshold: TAV = concentration/sensory threshold. Free taste-active amino acids play a key role in imparting taste. Thus, TAV greater than 1 for an amino acid indicates that it contributes to the overall food taste. The characteristic taste of TAPs from livestock liver is predominantly umami and sweet [47]. Zhou [77] predicted five representative TAPs from a mixture of chicken liver and fish bone paste, namely GVIHFQQQGSGPVK, LPEDLIK, GFFDRGASIVE, QPLDDDFVR, and VDVLEDKLK. All five peptides were predicted to have a sweet taste characteristic, and all except the first one also possessed an umami characteristic. According to the study by Zhou [77], the amino acids Asp, Glu, Lys, and Pro appeared most frequently in the predicted key TAPs, endowing the peptides with potential high-quality tastes such as umami, salt, and sweet taste. It should be acknowledged that part of the material in this study came from fish bone, which may have influenced the results for peptide and amino acid content. However, chicken liver still served as an important material basis for forming the aforementioned flavor-related amino acid profile. Therefore, this work by Zhou [77] still demonstrated the potential of chicken liver to produce TAPs, particularly in terms of sweet and umami taste.

As listed in Table 4, López-Martínez, Toldrá and Mora [47] indicated that in untreated pig liver (without ultrasound or enzymatic hydrolysis), the TAVs for umami amino acids Asp and Glu were 0.913 and 6.547, respectively. The TAVs for sweet amino acids Ser, Gly, Thr, and Ala were 0.596, 1.841, 0.157, and 2.397, respectively. The TAVs for bitter-sweet amino acids (Arg, Pro, Val, Lys, Tau, His, Tyr, Met) were 0.083, 0.227, 1.659, 2.769, 0.188, 0.320, 0.265, and 0.900, respectively. Bitter amino acid content also increased. It was found that in the unhydrolyzed sample, its potential taste profile was influenced by umami Glu, sweet Ala and Gly, and bitter-sweet Lys and Val. In the case of probe-ultrasound-pretreated raw pork liver sample (likely referring to a baseline or control sample), the influence of Met and His on taste was added. After hydrolysis with Alcalase for 120 min, the TAVs of almost all amino acids increased significantly. Sequential hydrolysis using Flavourzyme after Alcalase treatment resulted in tastes from Glu, Lys, and Ala similar to those from single-enzyme hydrolysis, while the tastes from His, Phe, and Met were significantly enhanced [47].

It is easily noticeable that in the processed samples, the taste activity of umami amino acid Glu is consistently very prominent compared to other amino acids. In almost all treatments covered in Table 4, the TAV for Glu is higher than that of other amino acids (except for bitter-sweet amino acids Val and Lys in the SH group), suggesting its umami characteristic is likely strong. Furthermore, after enzymatic hydrolysis, the TAVs for Val and Lys were also very prominent, both exceeding 20, while the highest TAV for bitter amino acids was 11.492. However, after hydrolysis, the TAVs for all six bitter amino acids were greater than 1, indicating the hydrolysate may possess a certain degree of bitterness. Methods for reducing bitterness will be discussed in Section 5.2.

López-Martínez, Toldrá and Mora [46] used Alcalase to treat porcine liver, reaching similar conclusions, and further discussed the impact of heat-cooking treatment on the flavor profile. Based on TAV results, the flavor characteristics of raw liver and Alcalase-hydrolyzed liver were similar to those of cooked liver, being particularly prominent in Glu, Ala, Val, Lys, His, Met, Ile, Leu, and Phe. This suggested their taste could be umami, sweet, sweet-sour (Val, Lys and Met), and definitely contained sulphurous and bitter notes. Zhou [77] also analyzed TAPs extracted from the mixture of chicken liver and fish bone paste, finding that the total content of umami and sweet amino acids accounted for 40.30% of the free amino acids, while bitter amino acids accounted for 38.08%. Among them, the TAVs for Glu and Asp were much higher than those of other amino acids.

In addition to umami, sweet, and bitter taste, Ahmad et al. [78] observed significant enrichment of 12 kokumi γ-glutamyl dipeptides after treating bovine liver with protease followed by glutaminase. Among the γ-glutamyl dipeptides, γ-Glu-Cys is the most potent agonist, followed by γ-Glu-Gln, γ-Glu-Ala, γ-Glu-Thr, γ-Glu-Leu, γ-Glu-Ile, γ-Glu-Ser, γ-Glu-Orn, γ-Glu-Met, γ-Glu-Asn, γ-Glu-Gly, and γ-Glu-Trp, demonstrating the potential of liver to produce kokumi peptides [78]. Taken together, although peptides from livestock liver inevitably possess bitter characteristics, the taste activity values of their free umami and sweet amino acids are prominently high. Liver-derived TAPs are also considered to have umami and sweet characteristics. Furthermore, livestock liver may have the potential to produce kokumi peptides.

### 5.2. Optimization Methods for Liver-Derived TAPs

Peptides in liver hydrolysates can undergo sensory optimization through the selection of appropriate methods to obtain improved sensory characteristics. Bitterness, as the primary limiting factor in taste perception, can be reduced or eliminated by means such as exopeptidase treatment, microbial fermentation, and the Maillard reaction [79]. In terms of taste perception, bitterness can also be mitigated or masked by umami through receptor interactions [80]. Therefore, although bitter peptides are released upon hydrolysis, the concurrent release of umami peptides may, to some extent, mitigate or mask the bitterness [46]. Different enzymatic treatments of the same sample lead to variations in free amino acid distribution, which in turn can affect sensory properties. Ultrasound-assisted enzymatic hydrolysis also influences the proportion of free amino acids and may enhance sensory characteristics. Due to space constraints, this section will not provide an extensive overview, as detailed explanations are provided in Section 3.1 and Section 3.3.

Furthermore, peptides are recognized as important flavor enhancers and precursors in the Maillard reaction. Studies have shown that the Maillard reaction can generate a variety of volatile compounds, forming specific aroma compounds, such as pyrazines, pyrazinones, thiazoles, and thiophenes. Maillard reaction products (MRPs) prepared from protein hydrolysates/peptides and carbohydrates can influence the flavor properties. For example, they can enhance umami and kokumi sensations, mask bitterness (or generate low bitterness), and exhibit a biphasic effect on human saltiness perception [81]. Wei et al. [82] coated a tasteless basal cat food with Maillard reaction products derived from chicken liver protein hydrolysate. They found specific compounds within these MRPs, namely nonanal (B2), 2-propylpyridine (H4), and 3-octen-2-one (E2). Furthermore, Wei, Xie, Muhoza, Liu and Song [82] indicated that a higher content of free amino acids could promote the formation of key flavor compounds in the subsequent Maillard reaction. Consequently, the cat food sample with the highest free amino acid content achieved the highest preference rate. This study demonstrates the potential of MRPs derived from animal liver protein hydrolysates to improve food palatability.

Both fermentation pretreatment and ultrasound pretreatment have also been demonstrated to promote the Maillard reaction. Appropriate fermentation pretreatment can enhance the content of key flavor compounds in MRPs and increase palatability by altering the amino acid composition. Chen, Cui, Li, Wang, Chen, Zhou and Xu [7] co-fermented chicken liver protein hydrolysate using Lactobacillus plantarum and Saccharomyces cerevisiae. Their results showed that in the resulting MRPs, this treatment simultaneously reduced bitter amino acid content (with decreases of 40.98% and 51.90% in the CLPHM [chicken liver protein hydrolysate subjected to MR] and FCLPHM [fermented CLPH subjected to MR] groups, respectively) and increased the content of umami/sweet amino acids (sweet amino acids increased by 33.98% and 11.18%, and umami amino acids increased by 34.39% in the FCLPHM group). The reduction in bitter amino acids and the increase in umami or sweet amino acids were more pronounced in the fermented groups compared to the non-fermented group, thereby optimizing the Maillard reaction precursors. Furthermore, during the subsequent Maillard reaction process, co-fermentation was found to increase the consumption of reducing sugars and amide groups, significantly elevating the content of key flavor compounds in the MRPs. Chen, Zou, Wang, Xiong and Xu [14] reported that MRPs subjected to ultrasound pretreatment showed no significant differences in bitterness, sweetness, or umami intensity as measured by an electronic tongue. However, the volatile compound content increased, and the astringency characteristic was reduced. This indicates that ultrasound pretreatment can also steer the Maillard reaction toward the production of substances with more desirable sensory profiles.

Notably, although the Maillard reaction endows foods with favorable sensory properties, it may simultaneously reduce the nutritional value of food and pose potential health risks. These adverse effects include the loss of essential amino acids and impaired bioavailability of nutrients, as well as the generation of potentially hazardous substances. A typical example is the formation of acrylamide, which arises from the reaction between asparagine and trace reactive reducing sugars in meat under high-temperature conditions.

## 6. Future Research Directions and Challenges

Current research still exhibits certain limitations, including inefficient prediction and screening of peptides, a lack of in vivo data on BPs, and insufficient breadth in studies on TAPs. Therefore, future research should employ artificial intelligence (AI) to assist in peptide prediction and screening, adopt strategies to enhance peptide absorption in the human body, and broaden the research scope to address these shortcomings, thereby making the high-value utilization of liver-derived peptides feasible.

(1) Although existing studies have isolated and verified certain bioactivities and flavor activities of liver-derived peptides, significant limitations remain. A prominent issue is the low efficiency in predicting peptide activity and screening for effective peptides. The prolonged duration of related pretreatment and instrumental analysis significantly extends the cycle of peptide screening and discovery. Consequently, there is an urgent need to develop new technologies to overcome the limitations of current methods [83]. This issue can be addressed through AI strategies. This approach encompasses several processes, such as basic data preparation, molecular feature representation, model construction and training, as well as evaluation and validation. The molecular feature representation step involves converting raw data into a format that can be understood and utilized by machine learning models. Model training and construction involve deep learning methods. For example, the convolutional neural network model BiteNetPp converts the three-dimensional spatial structure of proteins into a four-dimensional tensor representation. In the evaluation and validation process, preliminary screening is first conducted using the trained and optimized AI model. Subsequently, advanced computational screening techniques, such as molecular docking, are applied to enhance screening accuracy. Subsequent verification involves rigorous in vitro and in vivo studies to ensure that the identified active peptides (including TAPs) not only align with theoretical expectations but also demonstrate practical applicability [84].

(2) Beyond issues related to AI application, current liver-derived bioactive peptides face inherent limitations in vivo verification. The commercially produced bioactive peptides often suffer from insufficient quality stability, and their efficacy can be compromised by the digestive and metabolic processes within the human body [85]. Therefore, chemical modification and encapsulation technologies are commonly employed to enhance the digestive and metabolic stability of bioactive peptides [86]. Chemical modifications include D-amino acid or non-natural amino acid substitution, increasing molecular weight, cyclization, and N-/C-terminal modification or substitution. Encapsulation typically involves the use of microcapsules, liposomes, emulsions, polymer nanoparticles, and hydrogels for the delivery of bioactive peptides to resist degradation by bile salts, pancreatic enzymes, and acidic environments [86,87]. Among these, liposomes are particularly suitable as encapsulation materials due to their biocompatibility, non-toxicity, and amphiphilic nature [45]. In addition to the aforementioned technologies, permeation enhancers may also be utilized to increase the absorption of bioactive peptides. Their mechanisms of action include modulating tight junctions between epithelial cells or enhancing cell membrane permeability. Sodium caprate, bile salts, and chitosan have been demonstrated to fulfill both of the aforementioned functions [87]. Future research should fully consider the application of the aforementioned chemical modifications, encapsulation, and permeation enhancers to increase the absorption of liver-derived bioactive peptides in humans. Subsequently, bioactivity verification and enhancement should be conducted based on these strategies.

(3) Research on liver-derived taste-active peptides (TAPs) and bioactive peptides (BPs) is limited in scope. For TAPs, existing studies suffer from narrow coverage of animal species, inadequate systematic screening, and incomplete elucidation of functional mechanisms. As a high-value livestock and poultry by-product with great exploitation potential, liver can be utilized to prepare taste-active peptides [15]. Nevertheless, specialized research targeting liver-derived TAPs remains scarce. Most of the available literature focuses on flavor improvement of meat products via the Maillard reaction, while systematic investigations specifically on TAPs from livestock and poultry livers are scarce, leaving obvious research gaps regarding common species, such as duck, goose and sheep. Meanwhile, the screening and identification of highly potent liver-derived TAPs are insufficient, and the specific molecular mechanisms underlying their taste activity have not yet been clarified. Current mechanistic analyses mostly adopt research frameworks established for umami peptides from other sources, lacking dedicated investigations into the unique structure–activity relationships and taste action mechanisms of liver-derived TAPs. In terms of BPs, a lack of animal model data and clinical evidence is a prominent drawback. Only a small number of animal model studies have verified their antioxidant activity, while nearly no animal experiments have been conducted to confirm their other bioactivities. Furthermore, almost no clinical trials have been carried out to evaluate the bioactivities of liver-derived peptides. Accordingly, the reliability of current findings on the bioactivities of liver-derived peptides is restricted, and these results cannot fully demonstrate their efficacy in humans. To address the above limitations, future research should firstly expand the research scope of TAPs derived from livers of various livestock and poultry species to fill the research gaps across multiple animal species. Secondly, combined with the intrinsic physicochemical characteristics of liver-derived peptides, in-depth exploration of their structure–activity relationships and taste formation mechanisms should be performed. Additionally, more animal and clinical models should be established for liver-derived bioactive peptides to fully validate their functional effects in human bodies.

From the perspective of application prospects, liver-derived bioactive peptides and taste-active peptides possess high value for industrial food development. These peptides exhibit umami- and saltiness-enhancing taste properties, which can be utilized to develop low-sodium seasonings and improve the palatability of pet food [82]. Meanwhile, their inherent antioxidant and antibacterial activities make them suitable for the preservation of meat products, and they can also serve as alternatives to synthetic preservatives and antioxidants in other products to meet consumers’ demand for natural ingredients. Furthermore, their prominent antioxidant, anti-inflammatory and ACE-inhibitory (antihypertensive) activities provide high-quality raw materials for the research and development of functional health foods. The preparation of bioactive peptides from livestock and poultry livers can not only reduce processing waste, but also markedly raise the economic added value of livestock and poultry by-products. Despite promising application prospects, they face prominent commercialization barriers. From a technological perspective, it is difficult to achieve targeted enrichment of target peptides due to high heterogeneity of peptide sequences. Hydrophobic peptides inherently carry bitter taste defects, while debittering techniques add production costs and reduce bioavailability. With regard to regulations and safety, there is a lack of unified global regulatory standards for bioactive peptides applied in food products. Raw materials derived from animal viscera require comprehensive toxicological assessments covering heavy metals, pathogenic microorganisms and allergens. European and American authorities impose stringent approval requirements on health claims for visceral-source peptides, and efficacy verified by animal experiments cannot be directly converted into authorized functional claims on product labels [1]. From a market perspective, peptides tend to lose activity under pH fluctuations and thermal treatments during food processing and storage, and supporting technologies to maintain their stability in meat products and seasoning matrices remain immature. Future research may leverage artificial intelligence to screen low-bitter and high-activity peptides in a targeted manner, optimize low-cost encapsulation delivery systems, complete clinical safety data, and establish unified efficacy evaluation criteria, so as to advance the industrialized application of liver-derived peptides in food fields, such as low-sodium seasoning and antioxidant functional foods [84].

## Figures and Tables

**Table 1:** The protein contents of livers in common livestock and poultry.

Category	Liver Source	Protein Content (g/100 g)	References
Poultry	Chicken liver	19.1	[4]
	Goose liver	6.5	[4]
	Duck liver	22.7	[18]
Livestock	Porcine liver	18.0	[4]
	Buffalo liver	21.4	[19]
	Sheep liver	20.38	[20]
	Goat liver	20.32	[20]
	Donkey liver	22.6	[4]

**Table 2:** Main methods for extracting protein hydrolysates from livestock livers.

Methods	Sources	Methods	Results	References
Enzymatic hydrolysis	Porcine	Computer-simulated hydrolysis was performed using papain, bromelain, pepsin, and trypsin.	The levels of Glu, Asp, and Leu were the highest, accounting for 13.5%, 9.2%, and 9.7% of all the amino acids, respectively.	[9]
		Alcalase (50 °C, pH = 6), bromelain (40 °C, pH = 6), Flavourzyme (50 °C, pH = 5.5) and papain (37 °C, pH = 6), enzyme-to-substrate (E/S) ratio, 7 h	The proteolysis index was 82.56%, 70.37%, 63.55%, and 59.20%, respectively. Alcalase exhibited the highest hydrolysis efficiency (82.56%).	[28]
		Flavourzyme (50 °C, pH = 5.5), E/S ratio = 1:100, 4–10 h	Glu: 721.71 mg/100 g liver Lys: 751.71 mg/100 g liver Leu: 825.57 mg/100 g liver	[29]
		Pepsin, Alcalase, and trypsin were treated for 5 h under optimal conditions (manufacturer’s standard conditions); the degree of hydrolysis was determined.	MW < 3 kDa: 58%, 63%, 53%, MW > 10 kDa: 4%, 6%, 25%.	[30]
		Alcalase (50 °C, pH = 9), Neutrase (40 °C, pH = 7.5), papain (37 °C, pH = 6), Protamex (50 °C, pH = 7.5), pepsin (37 °C, pH = 3), and trypsin (37 °C, pH = 8), E/S ratio ranging from 1500 to 6000 U/g, 1–5 h.	Within 5 h of hydrolysis, Alcalase yielded the highest peptide content (51.55 mg/mL), while pepsin produced the lowest peptide content (7.33 mg/mL).	[6]
		Proteinase K, thermolysin, papain, bromelain and subtilisin, potential bioactivities were predicted via in silico analysis using the BIOPEP tool	Papain and subtilisin showed higher hydrolysis efficiency.	[31]
	Duck	Alcalase (50 °C, pH = 8), Neutrase (50 °C, pH = 8), papain (50 °C, pH = 7), E/S ratio = 1:100, 5 h	Alcalase showed the highest TCA-NSI value (55.62%), followed by papain (43.98%) and Neutrase (42.42%).	[32]
	Beef	Select one of the following pretreatment methods: No pretreatment, pepsin hydrolysis for 2 h, or ultrasound-assisted pepsin hydrolysis for 1–2 h; followed by hydrolysis with Flavourzyme (50 °C, pH = 5.5), E/S ratio = 1:100, 2 h	DH: no pretreatment 6.42%; pepsin: 18.8%; ultrasound-assisted enzymatic hydrolysis: DH = 21–25%	[10]
		Pepsin (37 °C, pH = 2), Alcalase (50 °C, pH = 8), Neutrase (50 °C, pH = 7), trypsin (50 °C, pH = 6.5), papain (50 °C, pH = 7.5), and Flavourzyme (50 °C, pH = 6.5), 4 h, 0.4%	Protein recovery: 18.3–37.3%; Alcalase showed the highest protein recovery, followed by papain.	[33]
		Papain (37 °C, pH = 6), Pepsin (37 °C, pH = 2.5), E/S ratio = 1:100, 3 h–24 h	Papain was the most effective enzyme for hydrolyzing meat proteins rich in Gly and Hyp.	[12]
	Bubalus bubalis	Proteinase-K (37 °C, pH = 8), E/S ratio = 1:200, pronase-E (37 °C, pH = 7–8), E/S ratio = 1:200, and ginger protease (60 °C, pH = 6–8), E/S ratio = 1:100, 4 h	DH: 55% (proteinase-K); 47.5% (pronase-E); 39% (ginger protease)	[34]
	Chicken	Alacalase (45 °C), 1.5%, 150 min	DH: 26.12%	[11,35]
	Goose	Pepsin (37 °C, pH = 3), 4 h, isolation and identification of bioactive peptides	Molecular weight of peptides < 3 kDa	[36]
Microbial fermentation	Porcine	Enzymatic hydrolysis groups: Alcalase (50 °C, pH = 8), papain (37 °C, pH = 6.5), pepsin (37 °C, pH = 3) Microbial group: Microbial suspension from *M. purpureus* (37 °C, pH = 5), 3–12 h	DH: Papain reached the maximum of 30.6% at 12 h; M3 showed the minimum of 0.6% at 3 h.	[37]
	Duck	*Bacillus subtilis* BNCC109047 was inoculated at concentrations of 2%, 3%, 4%, 5% and 6%, 37 °C, 12–60 h	/	[38]
	Chicken	Microbial group: *Pediococcus acidilactici* NCIM5368 (37 °C, pH = 4.2), 10%, 6–24 h Enzymatic hydrolysis group: Alcalase (45 °C), 1.5%, 150 min	DH: Enzymatic hydrolysis group 26.12%, fermentation group 14.3%	[35]
		*L. plantarum* and *S. cerevisiae* suspension at the ratio of 1:2 (30 °C, pH = 6.5), total concentration 2%, 48 h	Leu: 5.96; Glu: 5.51; Ala: 4.81 mg/g hydrolysate	[7]
Ultrasound-assisted enzymolysis	Chicken Liver (heart)	Ultrasound-assisted treatment (below 560 W, 20 kHz, 10–30 min), followed by papain treatment (65 °C, pH = 6.5), E/S ratio = 0.05–0.1, 75–150 min	Hydrolysis rate reached 99.64% at ultrasound 30 min + enzymatic hydrolysis 75 min (the highest).	[39]
	Goose	The mixture was treated at 0, 300 and 600 W for 30 min, followed by pepsin treatment (37 °C, pH = 2.0), E/S ratio = 1:100, 2 h	Particle size: control group 509.70 μm, 300 W group 313.70 μm, 600 W group 273.10 μm. The sulfhydryl group content of GLP decreased by 19.84% (300 W) and 29.67% (600 W), respectively.	[40]
	Beef	After treatment with alkaline protease for 5 h, ultrasound treatment (100–500 W, 20 min)	The proportion of 500–1000 Da faction increased by 13.8% after ultrasound treatment.	[8]
Ultrasound-assisted fermentation	Chicken	*Pediococcus pentosaceus*, *Lactobacillus plantarum*, and *Kombucha* (37 °C, 1%), 24 h, 560 W	The total essential amino acid content increased by 78.80%.	[41]

**Table 3:** Bioactivities and structural characteristics of bioactive peptides derived from livestock and poultry liver.

Activity	Source	Extraction Method	Results	Structural Characteristics	References
Antioxidant activity	Porcine	Hydrolysis with papain, bromelain, pepsin and trypsin	Free radical scavenging activity was 78% at 800 mg/mL. The Trolox equivalent values of FWG, MFLG and SDPPLVFG were 0.3, 0.7 and 4.6, respectively. 3T3 fibroblast antioxidant activity assay: FWG exhibited the highest antioxidant activity (approximately 50%) at 50 μmol/L.	FWG, MFLG and SDPPLVFVG	[9]
		Hydrolysis with Alcalase, bromelain, Flavourzyme and papain, respectively	APAAAI GPYSQLVDR showed negative correlations with DPPH, ABTS and FRAP (–0.523, –0.724 and –0.562, respectively). GLNQALVDL HALGSAR, ALFQDVQKPSDEWGK and LSGPQAGLGEYLFER were positively correlated with ORAC (0.743, 0.605 and 0.682, respectively). LGEHNIDVLEGNEQFINAAK showed strong positive correlations with ABTS, FRAP and ORAC (0.789, 0.592 and 0.619, respectively)	APAAIGPYSQLVDR, GLNQALVDLHALGSAR, ALFQDVQKPSQDEWGK, LSGPQAGLGEYLFER and LGEHNIDVLEGNEQFINAAK	[28]
		Hydrolyzed with Flavourzyme, followed by filtration using three pore sizes (5, 10, and 30 kDa)	ABTS value: 388.91 and 497.26 mg/100 g for 5 kDa and 30 kDa fractions, respectively. DPPH assay: The 30 kDa fraction exhibited the strongest antioxidant activity (970.06 μg Trolox/g) after 4 h.	The peptide fraction with the highest antioxidant activity was the 30 kDa component	[29]
		Hydrolyzed separately with pepsin, Alcalase, and trypsin	At a hydrolysate concentration (porcine liver hydrolysate-PLH) of 2 mg/mL produced by Alcalase, the DPPH radical scavenging rate was 79.02%, and the ABTS radical scavenging rate was 79.72%. In vitro ADH (alcohol dehydrogenase) and ALDH (acetaldehyde dehydrogenase) activation: the increments reached 89.99% and 79.16%, respectively. NTLPHPTAP showed the lowest binding energy with ADH (−0.88 kcal/mol) and low toxicity.	NTLPHPTAP	[30]
		Hydrolyzed separately with Alcalase^®^, papain, pepsin, or *Monascus* microbial suspension	DPPH radical scavenging: MPLH was the highest (63%), followed by PePLH (55%), and APLH and PaPLH (42% and 37%, respectively). APLH and PaPLH showed higher ferrous ion chelating ability than PePLH and MPLH. The most effective chelating ability was observed at 12 h. The reducing power of MPLH (0.65) was significantly higher than that of other hydrolysates (0.2–0.4).	Most APLH peptide fractions ranged in molecular weight from 500 to 4300 Da, similar to PaPLH. The total sulfhydryl amino acid contents: APLH19.4, PaPLH29.4, PePLH27.6, and MPLH25.7 mg/100 g sample The total aromatic amino acid contents: 3.75, 6.81, 6.88, and 5.28 mg/100 g sample.	[37]
		Samples were co-treated with papain and subtilisin	The liver peptide equivalent was 0.68 Trolox/mg	LK, KP and LKP	[31]
	Beef	Hydrolyzed with Flavourzyme, followed by pepsin hydrolysis or ultrasound-assisted treatment	ABTS: PUS2F reached 117.94 nmol TE/mg, followed by PUS1F and pre-treated with pepsin (PF) samples with values of approximately 100 nmol TE/mg. ORAC: PUS1F showed the highest activity (217.36 nmol TE/mg). RACI: PUS1F (ultrasound-assisted pepsin hydrolysis for 1 h) and PUS2F (ultrasound-assisted pepsin hydrolysis for 2 h) exhibited the highest values (close to 1). FRS values of SWGPGIP, GGGPWGNKGY and VGPGKWPGARN ranged from 0.525 to 0.558.	SWGPGIP, GGGPWGNKGY and VGPGKWPGARN	[10]
		Pepsin, alkaline protease, neutral protease, trypsin, papain, and flavor proteinase	DPPH radical scavenging rates of alkaline protease and papain groups were 21.3–47.3% and 19.9–43.4%, respectively, showing significant differences from other enzyme hydrolysates. Fe^2+^ chelating capacity: alcalase group was 24.4% higher than pepsin group. At 5 mg/mL, the reducing power of Alcalase-treated hydrolysate was 0.517, which was significantly higher than that of other hydrolysates (0.305–0.490).	The contents of acidic amino acids (Glu and Asp) in the Alcalase and papain groups were relatively higher.	[33]
		Hydrolyzed with papain and pepsin separately	After 24 h of hydrolysis, the hydrolysate showed the highest ABTS radical scavenging activity. Maximum DPPH radical scavenging rate: 92.56%. FRAP value: 17.88.	The content of free amino acids decreased after 24 h of hydrolysis.	[12]
		Alkaline protease hydrolysis followed by ultrasound treatment	Under 500 W ultrasound, the maximum values were as follows: ABTS (82.66%), DPPH (76.02%), Fe^2+^ chelating capacity (62.18%), and reducing power (1.2447%). Overall, ultrasound intervention increased the reducing power by 47.82–89.94%.	In the <500 Da fraction, the proportion of BLH was 48%, which decreased to 37–38% after ultrasound treatment. In the 500–1000 Da fraction, the proportion of BLH was 29%, and the molecular weight fraction increased by 13.8% after ultrasound treatment.	[8]
	Bubalus bubalis	Proteinase-K, pronase-E and ginger protease were used for separate treatment	DPPH: pronase-E showed the highest scavenging activity (82.4%, *p* < 0.05), followed by proteinase-K (61.6%) and ginger protease (43.7%).	/	[34]
	Duck	Alcalase	The subfraction exhibited the highest DPPH radical scavenging activity (45.55%), ABTS radical scavenging activity (77.68%), and hydroxyl radical scavenging activity (24.06%). Molecular docking revealed that hydrogen bonds with an average length of 2.19 Å were formed between peptides and DPPH radical. The viability of HepG2 cells treated with WDDMEKIWHH (500 μM) was 1.76 times higher.	GEHGDSSVPVWSGVN, HLDYYLGK, HLTPWIGK, DTYIRQPW, WDDMEKIWHH and MYPGIAD, 765.33–1525.68 Da	[32]
		Inoculated with *Bacillus subtilis*	At a concentration of 1.0 mg/mL, the DPPH radical scavenging rate was 87.46%, and the ABTS radical cation scavenging rate was 74.76%, which were higher than those of the injured group (49.32%). An oxidative damage model was established by stimulating HepG2 cells with 600 μmol/L H_2_O_2_.	MYGAVTPVK, NWEKIR, APGIIPR and RWWQLR, with molecular weights ranging from 723.45 to 981.51 Da.	[38]
	Chicken	Alcalase combined with *Pediococcus acidilactici* NCIM5368	DPPH radical scavenging activity: FCLH (96.14% at 24 h) showed stronger activity than ECLH (92.76%). ABTS radical scavenging activity: ECLH (19.29% at 24 h) was lower than FCLH (32.16%). Superoxide anion scavenging activity: FCLH (84.87–95.02%), ECLH (88.94%).	/	[35]
	Chicken	Papain treatment combined with ultrasound pretreatment	Papain treatment combined with 30 min of ultrasound pretreatment; E/S ratio = 0.1%, hydrolysis time = 75 min: Degree of hydrolysis (DH): 99.63%, ABTS: 503.9 mM TE. FRAP: 84.53 mM TE. Fe^2+^ chelating rate: 91.78%.	Ultrasound assistance promoted the production of low-molecular-weight peptides (58.6% for 1.7–1.35 kDa; 30.5% for <1.35 kDa) The hydrolysate was rich in Glu (16.7 mg/100 g of sample)	[39]
	Goose	Pepsin hydrolysis after ultrasound treatment	Hydroxyl radical scavenging: IC50 was decreased by 19.23% (300 W) and 11.26% (600 W). DPPH IC50 (300 W, 600 W) was 1.92, 0.80 and 1.28 mg/mL, respectively. At 5 mg/mL, the DPPH radical scavenging rates were 81.87%, 88.85% and 85.90%, respectively. At 5 mg/mL, the Fe^2+^ chelating capacity was increased by 30.57% (300 W) and 7.64% (600 W), respectively.	Ultrasound treatment significantly increased the β-sheet content of GLP and significantly decreased the α-helix and β-turn contents (*p* < 0.05).	[40]
Anti-inflammatory activity	Porcine	Alcalase	Optimal conditions: E/S ratio = 4913.25 U/g, hydrolysis time = 3.21 h. The NO inhibition rate of hydrolysate reached 24.81% at 29.90 mg/mL.	LFWFR, FFVFPR, DSFFPR, NSFFPR, NPLLFR, NFPLLK and DFPLLK	[6]
	Duck	Fermentation by *Bacillus subtilis* BNCC109047	LLVYPFPGPI and VIESPPEI significantly inhibited NO release from inflammatory cells, with inhibition rates of 47.24% and 56.32% at 100 μg/mL, respectively. TNF-α: VIESPPEI (P10) significantly reduced TNF-α release from inflammatory cells in a dose-dependent manner, with an inhibition rate of 42.48% at 100 μg/mL. IL-6: VIESPPEI exerted an inhibition rate of 96.73% against LPS-induced IL-6 release at 100 μg/mL.	The binding energy was lower than −20.93 kJ/mol. Peptides below 1500 Da accounted for 70.25%, while those above 3000 Da accounted for only 1.43%. DLTGIPPAP, ELKPTPEGDL, IDVSPDSPDHY, IYVDAVINH, LDSNLDLKF, LGEHNIDV, LVYPFPGPI, QTNLVPYPR, SLVYPFPGPIPN and VIESPPEI, with molecular weights ranging from 879.47 to 1299.68 Da.	[53]
		Alcalase and Flavourzyme	Compared with the LPS-induced group, the NO inhibition rates of groups F1–1, F1–3 and F1–4 were 55.46%, 45.30% and 37.49%, respectively. F1–1 subfraction effectively inhibited TNF-α release with an inhibition rate of 61.02%. VIESPPEI reduced TNF-α content in RAW264.7 cells in a dose-dependent manner, with an inhibition rate of 42.48% at 100 μg/mL. IDVSPDSPDHY exhibited the strongest inhibitory effect on IL-6 at low doses (27.04%).	Small peptides (<1500 Da) accounted for 70.25%. Peptide sequences: DLTGIPPAP, ELKPTPEGDL, IDVSPDSPDHY, IYVDAVINH, LDSNLDLKF, LGEHNIDV, LVYPFPGPI, QTNLVPYPR, SLVYPFPGPIPN, VIESPPEI (8–12 amino acid residues). The cysteine residue contents of LVYPFPGPI and VIESPPEI were 36.4% and 77.8%, respectively, and that of IDVSPDSPDHY was 62.5%.	[54]
Antihypertensive activity	Beef	Hydrolyzed with Flavourzyme, followed by pepsin hydrolysis, ultrasound or ultrasound-assisted pepsin hydrolysis for 1–2 h, with optional subsequent Flavourzyme hydrolysis	PF, PUS1F and PUS2F exhibited the highest inhibitory effects on ACE-I (angiotensin-converting enzyme inhibitory), with an inhibition rate of approximately 80%, ranging from 45% to 55%. dipeptidyl peptidase-IV (DPP-IV) inhibition: At 10 mg/mL, PF showed the highest inhibition rate (60.92%), with no significant difference between PUS1F and PUS2F. Neprilysin inhibition: PF and PUS1F showed the highest inhibition rate (87–89%) at 1 mg/mL.	>10 kDa: similar among CF, PF, PUS1F and US2F (approximately 64%). PUS1F decreased by 4%, and PUS2F decreased by 12%. 3–10 kDa: Decreased by 4.4–7.5%. Peptides smaller than 3 kDa accounted for approximately 30% in all samples, while this proportion reached 40% in PUS2F. Bioactive peptides for ACE and DPP-IV inhibition: QGAGPGGAGGF and YQGAGPGGGG	[10]
	Porcine	Hydrolyzed using endogenous proteases	The maximum ACE-I inhibitory rates of liver protein hydrolysates at pH 4.8 and 7.5 were 63.7% and 65.3%, with IC50 values of 0.886 g/L and 0.804 g/L, respectively.	YGLAAAVFTK, INYGGEIPK, GILAADESTGSIAKR, VAFTGSTQVGK, AGAGSATLSMAYAGAR, VGDKVLLPEYGGTK, AYQDQKPGTSGLR, IIPPGSGIIHQVNLEYLAR and AAWAFSR	[42]
	Bubalus bubalis	Proteinase-K, Pronase-E and ginger protease, respectively	Angiotensin I-converting enzyme (ACE-I) inhibitory activity: proteinase-K (50.1%), Pronase-E (48.8%), ginger protease (46.9%), and the untreated control sample (15.3%).	/	[34]
Lipid metabolism	Porcine	Alcalase	In palmitic acid (PA)-induced HepG2 cells: Lipid droplet accumulation was reduced, and the intensity of orange-red staining was significantly weakened in the PLPH (porcine liver protein hydrolysates) group (20 μg/mL). TC (total cholesterol) and TG (triglyceride) levels were decreased by 49.63% and 41.51%, respectively. The relative inhibition rates of FAS, ACC and CD36 mRNA were 50.33%, 16.39% and 48.17%, respectively. The expression of PPAR-γ mRNA was reduced by 24.83%.	LFWFR, FFVFPR, DSFFPR, NSFFPR, NPLLFR, NFPLLK and DFPLLK	[6]
Alcohol metabolism	Porcine	Treated separately with proteinase-K, pronase-E and ginger protease	In vitro ADL and ALDH activation: The activity increments of Alcalase hydrolysate reached 89.90% and 79.16% at 2.0 mg/mL, respectively. NTLPHPTAP showed the lowest binding energy with ADH (−0.88 kcal/mol). The Alcalase hydrolysate exhibited the most significant enhancement on ADH and ALDH activities, with the values of 75.484 ± 6.799% and 71.885 ± 4.25%, respectively.	After binding with NTLPHPTAP, the β-sheet content of ADH decreased, while the contents of α-helix, β-turn and random coil increased.	[30]
Antibacterial activity	Porcine	Hydrolyzed with Flavourzyme, followed by filtration using three pore sizes (5, 10, and 30 kDa)	Inhibition rate against *Brocothrix thermosphacta*: 83.3% at 4 h and 116.7% at 10 h for the 30 kDa fraction. The 10 kDa fraction showed a similar trend, with inhibition rates of 66.7% at 4 h and 100% at 10 h. The 30 kDa and 10 kDa fractions obtained at 10 h of hydrolysis inhibited *Listeria monocytogenes*, while other samples showed no such effect.	The peptide fraction (30 kDa) exhibited the highest antibacterial activity.	[29]
	Beef	Treated with papain and pepsin separately	The hydrolysate prepared by papain hydrolysis for 24 h showed stronger antibacterial activity against *Listeria monocytogenes* (17.5 mm) and *Salmonella enterica* subsp. enterica serovar Typhimurium (10.0 mm). The hydrolysate treated with pepsin only inhibited the growth of *Staphylococcus aureus* subspecies.	The antibacterial activity of liver hydrolysates against *Listeria monocytogenes* was negatively correlated with the contents of Glu, His, and Lys. There was a moderate linear correlation between them at *p* < 0.05.	[12]
Intestinal flora	Porcine	/	KITGLGVK reversed the abnormal levels of hepatic function markers (AST, ALT, TC, TG, TBA, TBIL) and ameliorated intestinal barrier damage. It normalized the levels of LPS, DAO, DLA, occludin, and claudin-1, reduced the abundance of Bacteroides and Sphingomonas, and restored the abundance of *Lactobacillus*, *Pediococcus*, and *Faecalibacterium*, thereby normalizing the levels of key intestinal metabolites.	KITGLGVK	[13]
Anti-anemia	Chicken	Treated with Alcalase or *Pediococcus acidilactici* NCIM5368	After being administered to Swiss albino female mice, the hemoglobin level recovered from less than 80% of the normal level under cyclophosphamide-induced anemia to 95% of the normal level. The total serum antioxidant activity of FCLH was 4.1–8.3, while that of ECLH ranged from 5.6 to 10.3. Antioxidants, iron, and various amino acids alleviated cyclophosphamide-induced anemia.	The sulfur-containing amino acid content of FCLH was higher than that of ECLH, which contributed to antioxidant activity. The amino acid group helped prevent alkylation and relieve anemia.	[11]
Anti-fibrosis activity	Chicken	Pepsin	TGF-β1 and SMAD4 were significantly downregulated in the CLHs or CNS groups compared with the TAA group (approximately 50–75% and 50–70%, respectively, as estimated from the graphical data in the study). In the TAA-treated group, the upregulated expression of Col1α and αSMA (*p* < 0.05) was significantly reduced by co-treatment with CLHs or CNS, with decreases by approximately 80% and 70%, respectively. The expression of αSMA protein in the TAA group was nearly 1.6 times higher.	/	[55]

**Table 4:** Taste activity values of protein hydrolysates from porcine liver.

Amino Acid	Taste Threshold mg/100 mL	No Ultrasound Pretreatment and No Alcalase Treatment	Bath Ultrasound Pretreatment Without Alcalase Treatment	Probe Ultrasound Pretreatment Without Alcalase Treatment	No Ultrasound Pretreatment, But with Alcalase Treatment	Both Bath Ultrasound Pretreatment and Alcalase Treatment
Asp	100	0.913	0.902	0.924	2.885	3.449
Glu	30	6.547	6.475	7.118	20.857	26.839
Ser	150	0.596	0.569	0.837	3.759	4.142
Gly	130	1.841	1.650	1.727	4.128	4.423
Thr	260	0.157	0.166	0.307	1.871	2.200
Ala	60	2.397	2.414	3.078	15.980	16.965
Arg	50	0.083	1.100	1.616	0.784	0.470
Pro	300	0.227	0.221	0.322	0.238	0.259
Val	40	1.659	1.603	1.414	21.160	21.200
Lys	50	2.769	2.655	3.238	21.506	24.553
Tau	1877	0.188	0.117	0.550	0.077	0.084
His	20	0.320	0.302	3.720	7.447	8.725
Tyr	96.6	0.265	0.242	0.420	3.843	4.502
Met	30	0.900	0.874	1.401	9.964	11.492
Ile	90	0.468	0.490	0.439	6.330	7.056
Leu	190	0.445	0.448	0.452	5.813	7.428
Phe	90	0.395	0.413	0.352	5.903	6.632
Trp	90	0.077	0.046	0.148	1.784	2.127

Note: Values in bold in the figure indicate a taste activity value > 1, meaning the amino acid flavor contributes to the overall flavor of the food, with higher values representing a greater contribution. Adapted from López-Martínez, Toldrá, and Mora [46] with permission of ACS.

## Data Availability

No new data were created or analyzed in this study. Data sharing is not applicable to this article.

## Abbreviations

The following abbreviations are used in this manuscript:

BPs	Bioactive peptides
TAPs	Taste-active peptides
DH	Degree of hydrolysis
NO	Nitric oxide
ACE	Angiotensin-converting enzyme
ECLH	Enzymatic chicken liver hydrolysate
FCLH	Fermented chicken liver hydrolysate
CLP	Chicken liver protein
E/S	Enzyme-to-substrate
AA	Amino acids
ROS	Reactive oxygen species
ACE-I	Angiotensin-converting enzyme inhibitory
ECE-I	endothelin-converting enzyme-I
PF	Pepsin followed by Flavourzyme
PUS1F	Ultrasound-assisted pepsin for 1 h followed by Flavourzyme
PLPHs	Porcine liver protein hydrolysates
TC	Total cholesterol
TG	Total triglyceride
DPP-IV	dipeptidyl peptidase-IV
SCFA	Short-chain fatty acid
FGF21	Fibroblast growth factor 21
AMPs	Antimicrobial peptides
GPCRs	G protein-coupled receptors
TRCs	Taste receptor cells
VFTD	Venus flytrap domain
CRD	Cysteine-rich domain
7-TMD	Seven-transmembrane domain
Gβγ	G-protein β and γ subunits
PLCβ2	Phospholipase C β2
PIP_2_	Phosphatidylinositol 4,5-bisphosphate
DAG	Diacylglycerol
IP_3_	Inositol 1,4,5-trisphosphate
TRPM5	Transient receptor potential cation channel subfamily M member 5
VFTM	Venus flytrap module
BU	Binding unit
SU	Stimulating unit
CaSR	Calcium-sensing receptor
MRPs	Maillard reaction products
AI	Artificial intelligence
